# Estrogen receptor beta enhances chemotherapy response of GBM cells by down regulating DNA damage response pathways

**DOI:** 10.1038/s41598-019-42313-8

**Published:** 2019-04-16

**Authors:** Mei Zhou, Gangadhara R. Sareddy, Mengxing Li, Jinyou Liu, Yiliao Luo, Prabhakar Pitta Venkata, Suryavathi Viswanadhapalli, Rajeshwar R. Tekmal, Andrew Brenner, Ratna K. Vadlamudi

**Affiliations:** 1 Department of Obstetrics and Gynecology, University of Texas Health San Antonio, San Antonio, TX 78229 USA; 2 Department of Gastroenterology, The Second Xiangya hospital, Central South University, Changsha Shi, Hunan 410008 P. R. China; 3 Department of Respiratory Medicine, Xiangya hospital, Central South University, Changsha Shi, Hunan 410008 P. R. China; 4 Department of Oncology, The Second Xiangya hospital, Central South University, Changsha Shi, Hunan 410008 P. R. China; 5 Department of General Surgery, Xiangya Hospital, Central South University, Changsha Shi, Hunan 410008 P. R. China; 6 Hematology & Oncology, University of Texas Health San Antonio, San Antonio, TX 78229 USA; 7 Mays Cancer Center, University of Texas Health San Antonio, San Antonio, TX 78229 USA

## Abstract

Glioblastoma (GBM) is the most commonly diagnosed brain tumor that exhibit high mortality rate and chemotherapy resistance is a major clinical problem. Recent studies suggest that estrogen receptor beta (ERβ), may function as a tumor suppressor in GBM. However, the mechanism(s) by which ERβ contributes to GBM suppression and chemotherapy response remains unknown. We examined the role of ERβ in the DNA damage response of GBM cells, and tested whether ERβ sensitizes GBM cells to chemotherapy. Cell viability and survival assays using multiple epitope tagged ERβ expressing established and primary GBM cells demonstrated that ERβ sensitizes GBM cells to DNA damaging agents including temozolomide (TMZ). RNA-seq studies using ERβ overexpression models revealed downregulation of number of genes involved in DNA recombination and repair, ATM signaling and cell cycle check point control. Gene set enrichment analysis (GSEA) suggested that ERβ–modulated genes were correlated negatively with homologous recombination, mismatch repair and G2M checkpoint genes. Further, RT-qPCR analysis revealed that chemotherapy induced activation of cell cycle arrest and apoptosis genes were attenuated in ERβKO cells. Additionally, ERβ overexpressing cells had a higher number of γH2AX foci following TMZ treatment. Mechanistic studies showed that ERβ plays an important role in homologous recombination (HR) mediated repair and ERβ reduced expression and activation of ATM upon DNA damage. More importantly, GBM cells expressing ERβ had increased survival when compared to control GBM cells in orthotopic GBM models. ERβ overexpression further enhanced the survival of mice to TMZ therapy in both TMZ sensitive and TMZ resistant GBM models. Additionally, IHC analysis revealed that ERβ tumors had increased expression of γH2AX and cleaved caspase-3. Using ERβ-overexpression and ERβ-KO GBM model cells, we have provided the evidence that ERβ is required for optimal chemotherapy induced DNA damage response and apoptosis in GBM cells.

## Introduction

Glioblastoma (GBM) is one of the most commonly diagnosed and aggressive form of primary malignant brain tumors in adults^1,2^. GBM is also among the most deadly neoplasms associated with worst 5-year overall survival (OS) rates amid all human cancers^3^. Standard treatment for GBM consists of surgically excising the tumor, in conjunction with external radiation therapy (XRT), and adjuvant chemotherapy with temozolomide (TMZ)^4,5^. However, developing resistance to XRT and chemotherapy is a major clinical problem^6,7^. While the mechanisms that contribute to therapy resistance in GBM are elusive, it is important to identify the mechanisms that would improve the patient’s response to current GBM treatment plans. Epidemiologic evidence suggests that estrogen plays a tumor-suppressive role on brain tumors^8,9^ and potentially plays a protective role in GBM progression^10,11^.

The biological effects of 17β-estradiol (E2) are mediated through both estrogen receptors (ER), ERα and ERβ. Despite extensive sequence and biochemical similarities, these ER subtypes have distinctly unique biological functions. For example, ERβ exhibits antitumor activity, a trait that is not exhibited by ERα^12^. Several studies have shown that overexpression of ERβ reduces cell proliferation and the knockdown of ERβ enhances cell proliferation in cancer cells^13,14^. As transcription factors, ERα and ERβ share many target genes; however, ERβ activates a unique set of genes^15,16^ via its direct DNA binding or its interactions with other transcription factors^15,17^. Recent studies showed GBM cells uniquely express ERβ^18^ and using knock out models it was demonstrated that ERβ has tumor suppression function in GBM^19^. However, the mechanism(s) by which ERβ promotes tumor suppression in GBM is poorly understood.

Recent studies have shown that ERβ alters the chemo-sensitivity of breast cancer cells^20^. Concurrently, ERβ agonists affect the sensitivity of malignant pleural mesothelial cells to cisplatin toxicity^21^ and the inhibition of ERβ, increases DNA repair, which in turn contributes to developing cisplatin resistance in medulloblastoma cells^22^. Our earlier and other *in vitro* studies have shown that ERβ agonists increases the sensitivity of GBM cells to chemotherapeutic agents that are currently used such as, TMZ and lomustine^23,24^. However, the significance and comprehension of *in vivo* mechanisms by which ERβ affects chemotherapy response in GBM cells and its molecular mechanisms are not fully understood.

In this study, we examined the mechanisms by which ERβ sensitizes GBM cells to standard chemotherapy. RNA-seq studies discovered that ERβ modulated several genes that are involved in DNA recombination, repair, and ATM signaling. Using *in vitro* assays, we provided evidence that ERβ sensitizes GBM cells to carboplatin, cisplatin, lomustine and TMZ treatments. Chemotherapy induced apoptosis and cell cycle arrest genes were attenuated in ERβ-KO cells. Using xenograft models, we have provided evidence *in vivo* demonstrating the tumor suppressor potential of ERβ and that ERβ sensitizes GBM to TMZ therapy. Our results suggest that ERβ is required for optimal chemotherapy induced DNA damage response and apoptosis in GBM cells.

## Results

### ERβ modulate DNA damage response pathways in GBM cells

**Figure 1:**
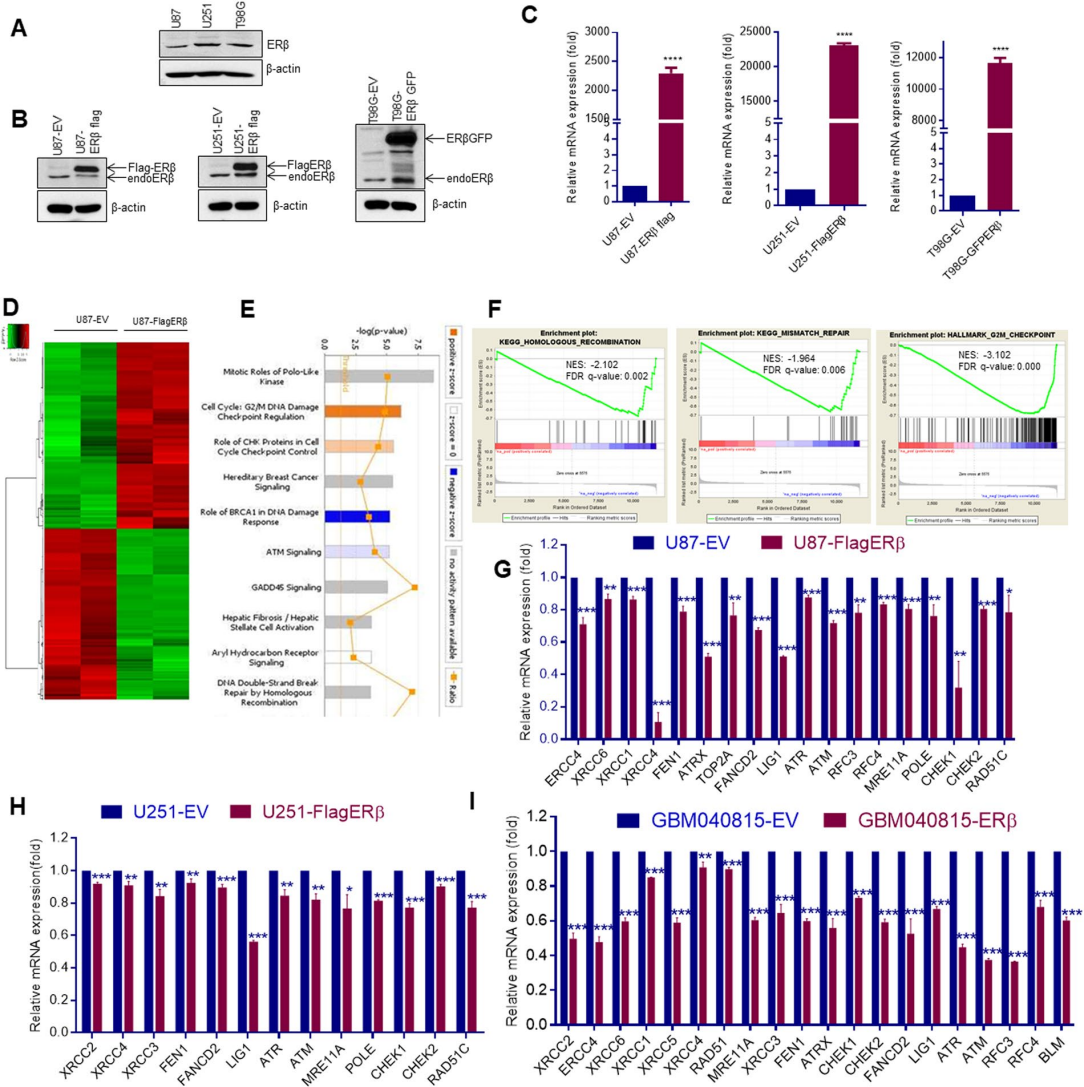
RNA-seq analysis of transcriptional changes induced by ERβ in GBM cells. (**A**) Western blot analysis of total lysates from three GBM models using validated ERβ monoclonal antibody (PPZ0506, Perseus proteomics). (**B**) Western blot analysis of ERβ in three GBM models that express FlagERβ (U87, U251) or ERβGFP (T98G). (**C**) Validation of ERβ expression levels using RT-qPCR in empty vector or FlagERβ or ERβGFP expressing cells. Total RNA was isolated from the U87-empty vector (EV), and U87-ERβ cells and subjected to RNA sequencing. (**D**) Heat map showing genes differentially expressed between the two groups. (**E**) Top pathways modulated in U87-ERβ cells compared to U87-EV cells analyzed by IPA. (**F**) GSEA testing correlation of ERβ -modulated genes with signatures of the homologous recombination, mismatch repair and G2M check point. (**G**–**I**) The selective genes were validated using RT-qPCR in empty vector or FlagERβ expressing U87, U251 and GBM040815 cells. Data are shown as mean ± SE. *p < 0.05; **p < 0.01; ***p < 0.001.

Western blot analysis using validated ERβ antibody showed that all three GBM models express ERβ, however, at lower levels (Fig. 1A). To test the significance of ERβ, we generated GBM model cells that overexpress epitope tagged ERβ. We have used two different epitope tags (Flag and GFP). U87- and U251-ERβ model cells express FlagERβ while T98G-ERβ model cells express ERβGFP. Flag tag is at the N terminus of ERβ after ATG, while GFP tag is at the C terminus of ERβ before the stop codon. As shown in Fig. 1B, U87-FlagERβ model cells have threefold overexpression, U251-FlagERβ model cells have fourfold overexpression and T98G-ERβGFP models have fivefold overexpression compared to the levels of endogenous ERβ. We also confirmed expression of endogenous ERβ in control cells and ERβ overexpressing GBM models using RT-qPCR (Fig. 1C). To determine the mechanism of ERβ mediated tumor suppression, we performed RNA-seq analysis using U87 empty vector (EV), and U87-FlagERβ cells. Overall, 1001 genes (1.5 fold change over control with adjusted p-value < 0.05) were expressed differentially in ERβ cells; of which 477 genes were downregulated, and 524 genes were upregulated. The RNA-sequencing results were deposited in the GEO database under accession number GSE121332. The differentially expressed genes are shown between the groups in the heat map (Fig. 1D). The ingenuity pathway analysis (IPA) of differentially expressed genes between U87-EV vs U87-ERβ cells revealed that the ERβ–modulated genes were related to DNA damage check point regulation, DNA damage response, DNA repair, ATM signaling pathways and cell cycle (Fig. 1E). Further, GSEA revealed that ERβ regulated genes showed negative correlation with homologous recombination, mismatch repair, and G2M checkpoint gene sets (Fig. 1F). RT-qPCR analysis using established GBM cells (U87, U251) and patient derived GBM cells (GBM040815), confirmed that genes related to DNA damage response were significantly down regulated in ERβ overexpressing cells compared to control cells (Fig. 1G–I). These results, as a whole, suggest that in GBM cells, ERβ modulated the DNA damage response pathways.

### ERβ enhances chemotherapy response in GBM cells

**Figure 2:**
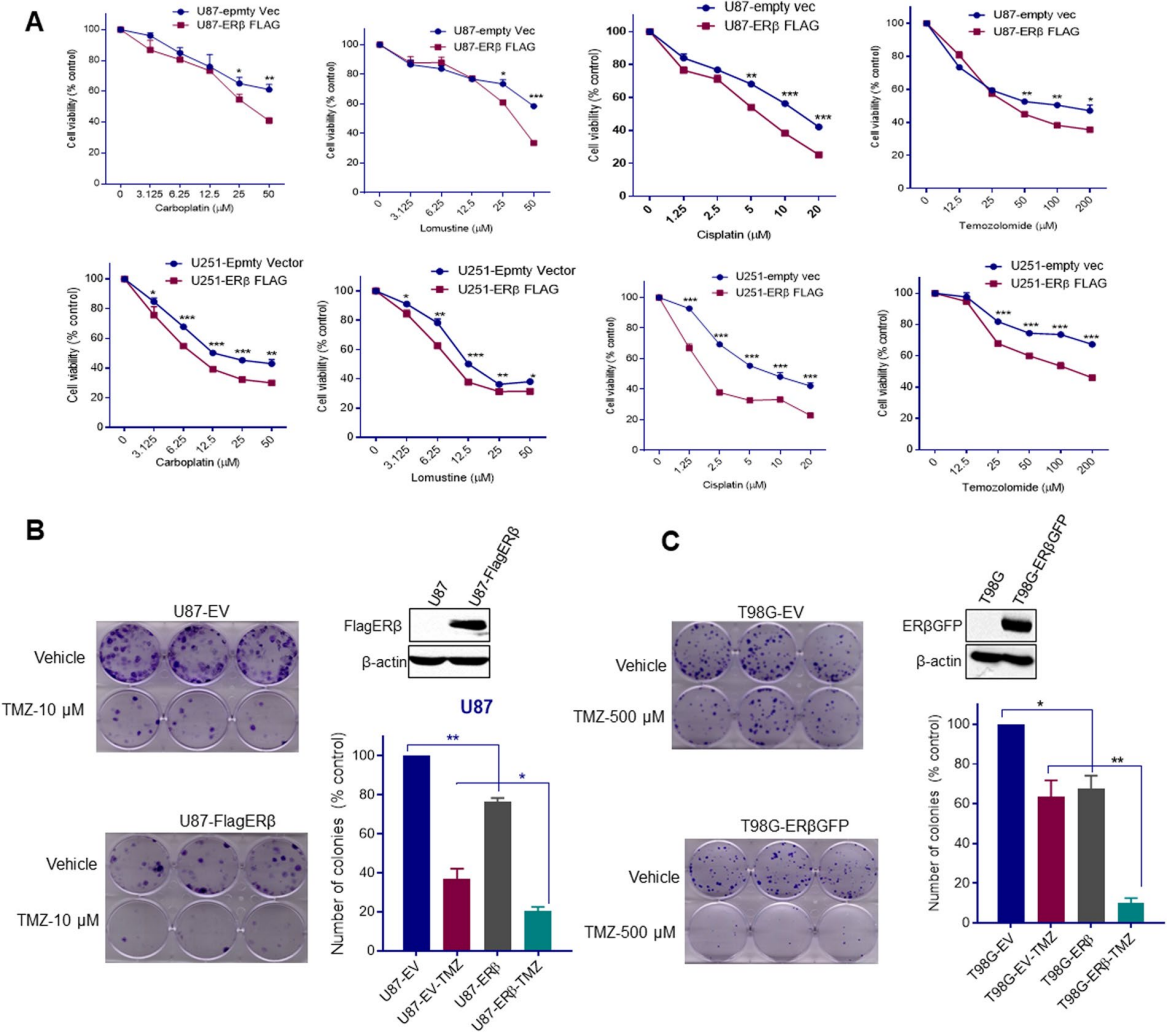
ERβ sensitizes GBM cells to chemotherapy. (**A**) Equal number of U87-EV, U87-ERβ or U251-EV, U251-ERβ cells were treated with different doses of chemotherapeutic drugs (carboplatin, lomustine, cisplatin, temozolomide) and cytotoxicity was evaluated using an MTT assay. Equal number of U87-EV, U87-FlagERβ (**B**) or T98G-EV, T98G-ERβGFP (**C**) cells were treated with TMZ for 3 days and after 14 days and the number of colonies were determined ERβ expression was confirmed by Flag or GFP epitope antibody using western blotting (right panel). Data are represented as mean ± SE. *p < 0.05; **p < 0.01; ***p < 0.001.

Since, ERβ overexpression attenuated the DNA damage response genes, we further examined the effect of ERβ expression on the response of GBM cells to various genotoxic agents commonly used for treating GBM (TMZ, lomustine, cisplatin and carboplatin). We used cell viability assays and tested using two GBM models (U87, U251) that stably express the empty vector (EV) or ERβ vector. ERβ expressing GBM cells exhibited enhanced cytotoxicity to these drugs compared to vector transfected cells (Fig. 2A). Further, in clonogenic survival assays, we found that ERβ expressing -U87 and -T98G cells showed significantly reduced colonies upon TMZ treatment compared to vector transfected cells (Fig. 2B,C). Collectively, these results provide the evidence that ERβ signaling has the potential to enhance chemotherapy response in GBM cells.

### ERβ is needed for optimal activation of chemotherapy induced apoptosis and cell cycle arrest genes

**Figure 3:**
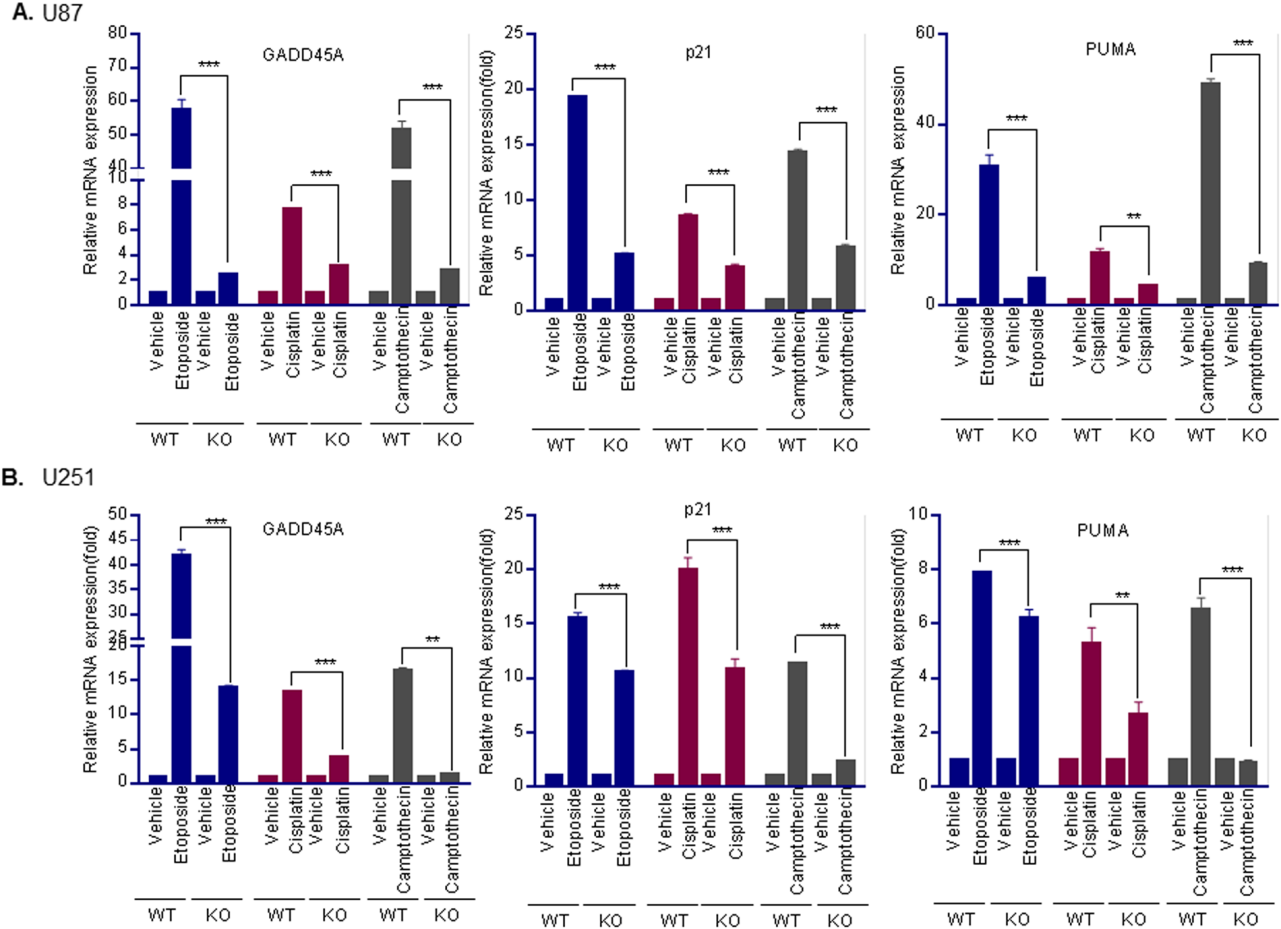
ERβ is essential for optimal activation of chemotherapy induced DDR genes. U87-WT, U87-ERβ KO (**A**) or U251-WT, U251-ERβ KO (**B**) cells were treated with etoposide (10 µM for 48 h), camptothecin (100 nM for 48 h) or cisplatin (5 µM for 48 h). RNA was isolated and the status of cell cycle arrest and apoptosis genes p21, puma and gadd45a was analyzed by qRT-PCR analysis. Data are shown as mean ± SE. **p < 0.01; ***p < 0.001.

To further confirm the role that ERβ plays in DNA damage response, we have used U87- ERβ-KO and U251-ERβ-KO cells that were recently generated using CRISPR/Cas9 system^19^. We treated U87-ERβ-KO, U251-ERβ-KO, and isogenic control cells with genotoxic agents including etoposide, cisplatin and camptothesin and measured the expression of DNA damage response genes that are involved in cell cycle arrest and apoptosis such as *p21, puma, and gadd45a* using RT-qPCR. Results showed that all three genes were significantly induced following treatment with genotoxic agents, however, the induction of these genes was significantly attenuated in ERβ-depleted cells in comparison to the control cells (Fig. 3A,B). These results suggest that ERβ plays an essential role in chemotherapy induced apoptosis and cell cycle arrest in GBM cells.

### ERβ modulate HR repair pathway by modulating ATM axis

**Figure 4:**
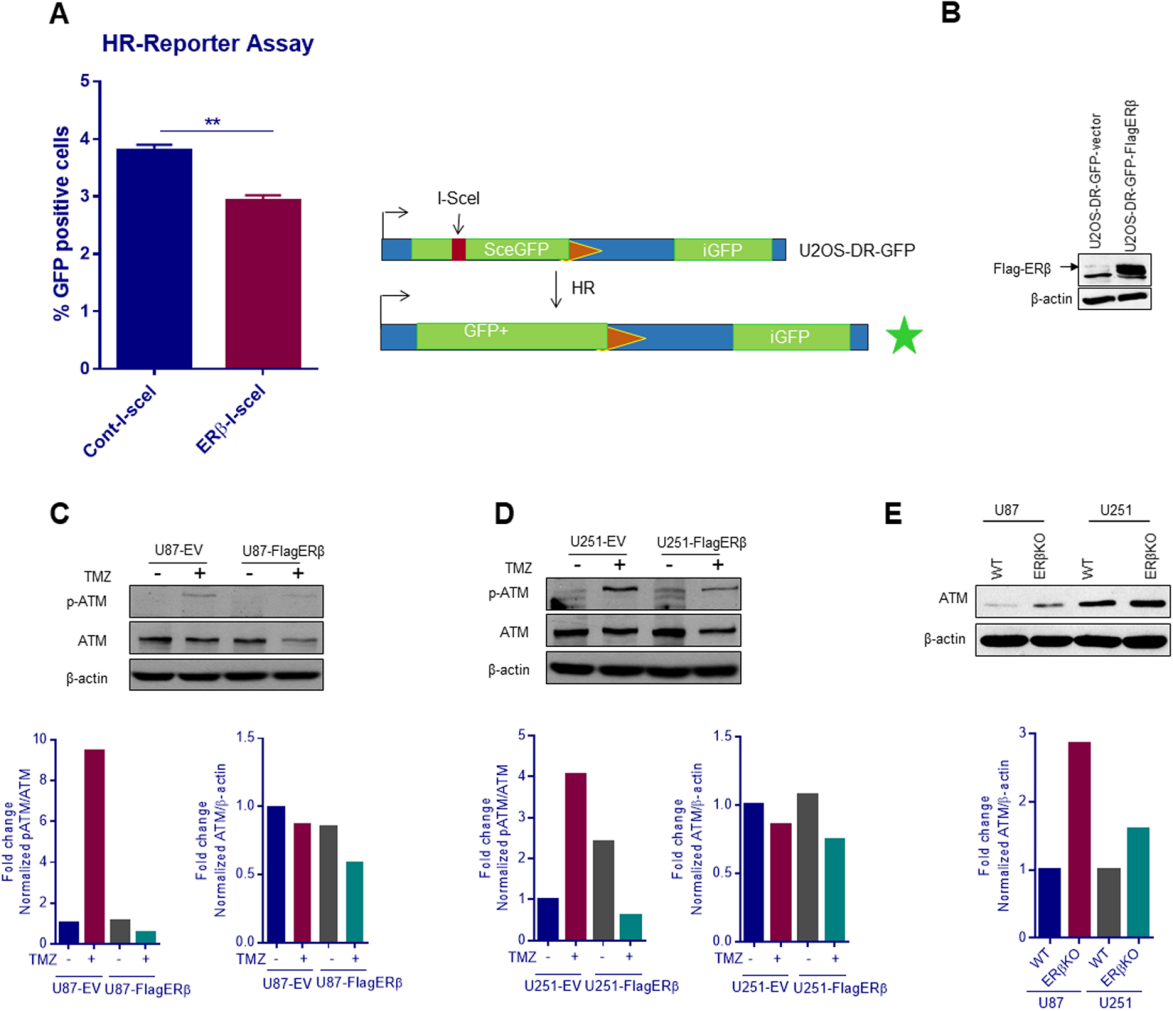
ERβ alters HR pathway by modulating ATM signaling axis. (**A**) U2OS DR-GFP or U2OS DR-GFP-FlagERβ cells were electroporated with 2 μg of pCBASce plasmid (I-SceI expression vector). Cells were harvested 2 days later and subjected to FACS analysis to determine the % GFP-positive cells resulting from HR repair of the I-SceI induced DSB. (**B**) ERβ expression was verified by Western blotting. U87-EV, U87-FlagERβ (**C**) or U251-EV, U251-FlagERβ (**D**) cells were treated with either vehicle or TMZ (25 μM for 48 h) and the status of phosphorylation of ATM was analyzed by Western blotting. (**E**) The expression levels of ATM was determined in U87-WT, U87-ERβKO, U251-WT and U251-ERβKO cells using western blotting. Band intensities were quantitated and plotted as histogram (bottom panels). **p < 0.01.

Since our RNA-sequencing results using GBM models indicated that ERβ down regulated the genes involved in homologous recombination (HR) pathway, we proceeded to determine whether ERβ played a role in HR pathway. We tested this potential role by assaying HR using U2OS cells stably integrated with direct repeat green fluorescent protein (DR-GFP) reporter plasmid^25^. In this system, the percentage of GFP reconstitution following a double strand break (DSB) induced by I-SceI endonuclease is a measure of DSB repair by HR. ERβ expression significantly downregulated the percentage of GFP reconstitution, indicating that ERβ may play a role in HR (Fig. 4A,B). To further demonstrate the importance of ERβ in DNA repair pathway, we tested whether phosphorylation of ATM was altered with or without TMZ treatment. Western blot results showed a substantial decrease in the activation of ATM in ERβ expressing cells compared to vector expressing cells (Fig. 4C,D). Further, ERβ expressing cells also showed decreased levels of ATM. Conversely, ERβ knockout cells had higher levels of ATM compared to control cells (Fig. 4E). Collectively, these results indicate that ERβ attenuates the HR pathway by modulating ATM signaling axis and repair.

### ERβ increases TMZ induced DNA damage

**Figure 5:**
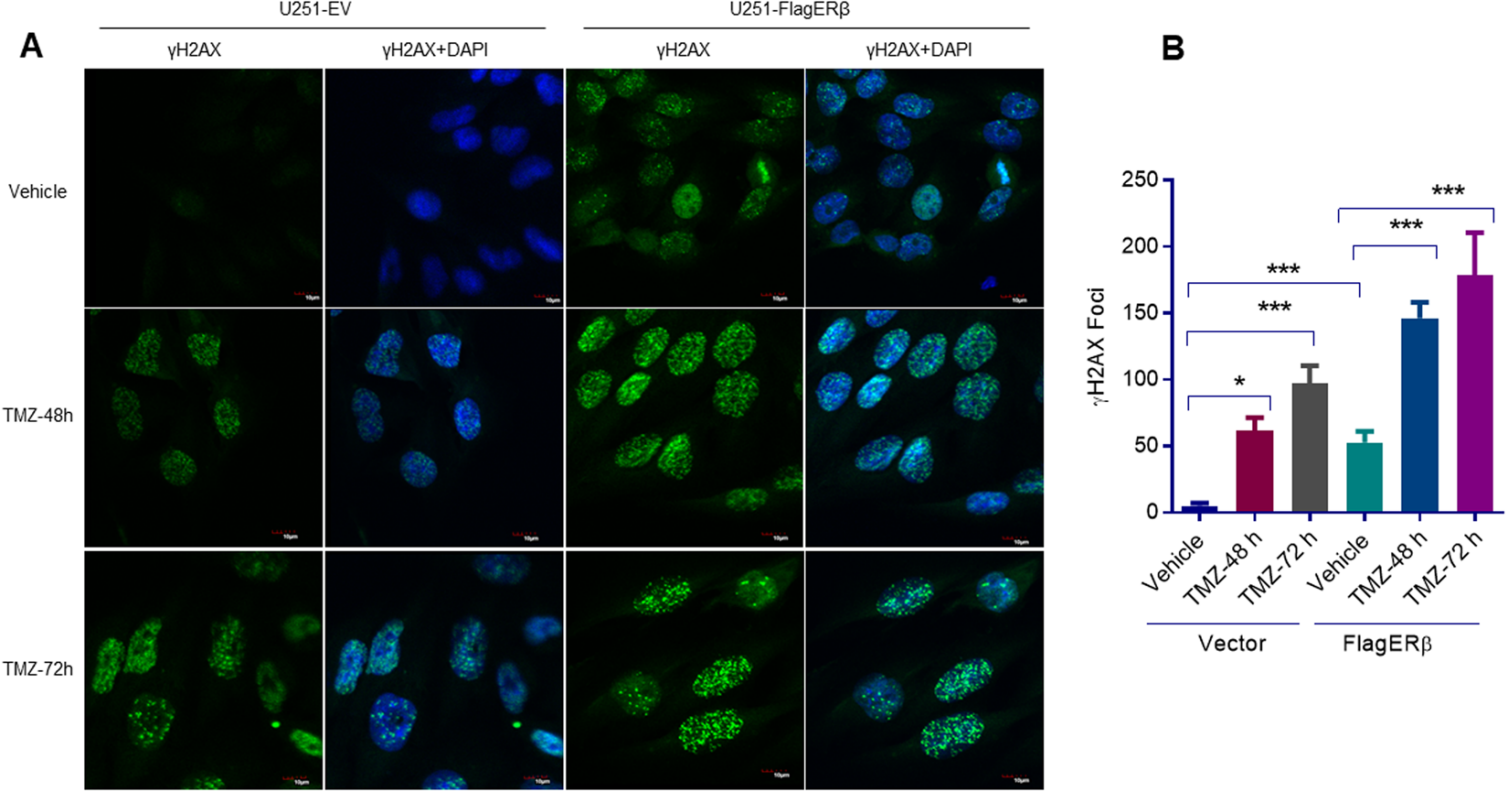
ERβ amplifies TMZ induced DNA damage in GBM cells. U251-EV, or U251-ERβ expressing cells were treated with either vehicle or TMZ for 48 hours and 72 hours and the γH2AX foci was captured using confocal microscope (**A**) and the number of foci was calculated and plotted as histogram (**B**). Data are represented as mean ± SE. *p < 0.05; ***p < 0.001.

The earliest event of DNA damage is the phosphorylation of H2AX and this response is observed within 30 minutes^26,27^. DNA damage is usually resolved within minutes to hours after the disappearance of γH2AX foci. Presence of prolonged γH2AX foci indicates unrepaired DNA and eventually leads to the induction of apoptosis. Since our RNA-seq experiments indicated the downregulation of DNA repair proteins, we further examined whether ERβ amplifies the TMZ induced DNA damage in GBM cells. Increased basal levels of γH2AX foci signaling was seen in FlagERβ expressing cells compared to control cells confirming the ability of exogenously expressed FlagERβ to suppress DNA damage response. Further, treatment of GBM cells with TMZ (for 48 h and 72 h) resulted in elevated levels of γH2AX foci compared to control cells; this effect was significantly enhanced in FlagERβ expressing U251 cells (Fig. 5A,B) Collectively, these results suggests that ERβ enhances the DNA damage induced by TMZ treatment.

### ERβ overexpression increased the survival of tumor-bearing mice upon chemotherapy

**Figure 6:**
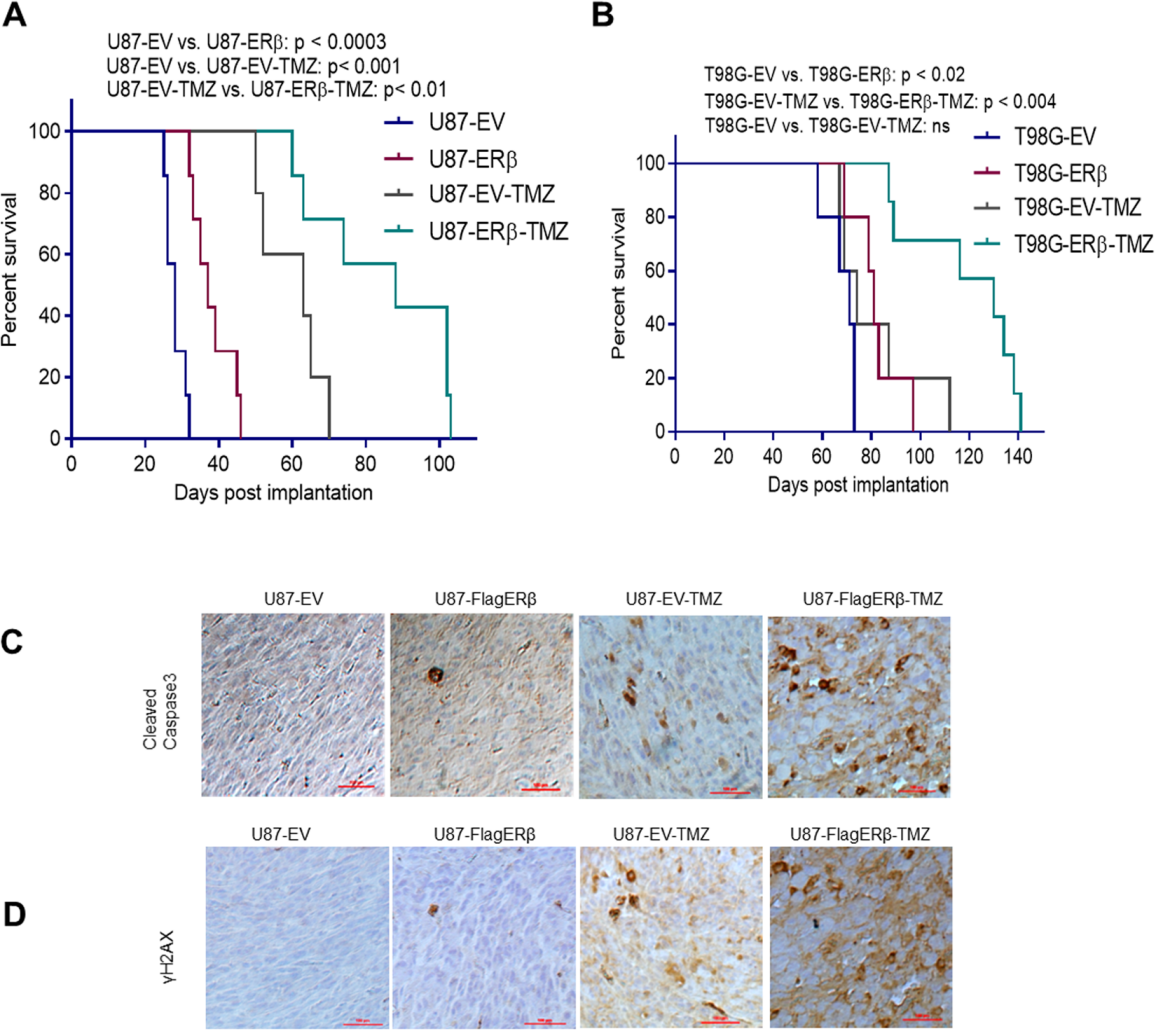
ERβ expression increase mice survival to TMZ treatment in orthotopic GBM models. (**A**) Mice were implanted with U87-EV or U87-FlagERβ (**A**) and T98G-EV or T98G-ERβGFP (**B**) cells orthotopically in the right cerebrum. After the tumor establishment, mice were treated with vehicle or TMZ and the survival of the mice was plotted using Kaplan-Meier curve. (**C**,**D**) Mice brains were collected, fixed in formalin, and processed for immunohistochemical staining for cleaved caspase-3 and γH2AX.

Next, we determined whether ERβ expression could sensitize GBM to TMZ treatment and improves the mice survival using *in vivo* orthotopic models. For this experiment we used, both TMZ sensitive U87 and TMZ resistant T98G cells. The results showed that the expression of ERβ significantly increased the survival of U87, as well as T98G tumor bearing mice compared to empty vector cells (Fig. 6A,B). Further, expression of ERβ significantly increased the survival of TMZ-sensitive U87 tumor bearing mice to TMZ treatment in comparison to vector control cells (Fig. 6A). More importantly, expression of ERβ also resulted in a significant increase in the survival of TMZ resistant T98G tumor bearing mice to TMZ therapy when compared to vector control cells (Fig. 6B). We further examined the levels of cleaved caspase3 and γH2AX in U87-tumors treated with vehicle or TMZ. IHC analysis of tumor sections revealed that TMZ treated ERβ expressing tumors had more cleaved caspase3 and γ H2AX positive cells than TMZ treated vector expressing tumors (Fig. 6C,D). These results demonstrated the ERβ possessed tumor-suppressing functions in GBM and has the potential to sensitize TMZ sensitive- and TMZ resistant- GBM cells to TMZ treatment.

## Discussion

Estrogen plays a crucial role in the differentiation of neuronal cells^28^. The tumor suppressor functions of ERβ are reported in many cancer models. Recently studies from our, and other labs have shown that GBM cells uniquely express ERβ and therefore, has the potential to function as a tumor suppressor^18^. However, the mechanisms that contribute to ERβ mediated tumor suppression remain elusive. A bottleneck to study the mechanism of ERβ is in part due to the lack of quality antibodies. To overcome this problem, we generated epitope tagged ERβ expression GBM models and studied their mechanisms. We also confirmed mechanism using ERβ-KO cells. Our results showed that 1) ERβ modulates a number of genes involved in DNA recombination and repair, 2) ERβ sensitizes GBM cells to chemotherapy drugs, 3) ERβ- KO attenuates chemotherapy induced apoptosis and cell cycle arrest genes, 4) ERβ attenuates HR repair by modulation of ATM signaling and 5) using a xenograft model, provided evidence *in vivo* that ERβ sensitizes GBM to chemotherapy. This data supports that ERβ is essential for optimal chemotherapy induced DNA damage response, as well as apoptosis in GBM cells.

Published genome-wide studies suggest that ERα and ERβ potentially activate different sets of genes, and the effects of ERβ with other transcription factors (AP1, SP1, NF- κB, and KLF5) can be non-classical via its interactions^15,17,29^. Our RNA-seq analysis revealed that ERβ modulated several unique pathways including those involved in DNA damage response. Further GSEA results also demonstrated that ERβ-modulated genes were negatively correlated with the homologous recombination and repair. In conjunction with mechanistic studies, we have shown that ERβ reduced the activation of ATM signaling in TMZ treated GBM cells compared to control cells. Collectively, our results discovered that ERβ plays an important role in modulation of chemotherapy response and its status may have important implications in chemotherapy response.

ERβ is shown to play a role in DNA damage response via Breast Cancer Associated Gene 2 (BCA2). ERβ agonist Erb-041 promotes the reduction of chromatin-bound BCA2 leading to an increased level of chromatin-bound γH2AX upon UVC irradiation. This indicates the important role of ERβ in regulating DNA damage response^30^. In breast cancer models, ERβ impairment of DNA damage response involves BRCA1 downregulation and caspase-2 activation which results in mitotic catastrophe and decreased cancer cell survival^31^. Future studies examining the molecular mechanism of ERβ signaling on DNA damage response in GBM progression will be useful in maximizing treatment opportunities for this deadly cancer.

Epidemiological studies suggest that hormone replacement treatment has protective effects against colorectal cancer. Estradiol regulates mismatch repair gene expressions via ERβ in colorectal cells^32^. ERβ-mediated nuclear interaction between IRS-1 and Rad51 is shown to inhibit HR directed DNA repair in medulloblastoma^33^. ERβ-agonist Erb-041 potentiated BCA2 dissociation from chromatin and co-localization with Rad51; this resulted in the inhibition of HR repair^30^. Our previous studies using ERβ-agonist LY500307 also showed suppression of  DNA repair pathways^23^. Here, using ERβ overexpression and under expression, we provided further evidence that ERβ has the ability to suppress DNA recombination and repair pathways. Specifically, our results support that ERβ regulates HR pathway of DNA repair by modulating ATM expression and functions. The ability of ERβ to suppress DNA repair is an important attribute of GBM suppression and loss of this function potentially reduces chemotherapy response.

Earlier studies using colon cancer cells showed that when ERβ is overexpressed, it may induce cell apoptosis and anti-proliferation by increasing p53 signaling^34^. ERβ alters the chemo sensitivity of luminal breast cancer cells by regulating p53 function^20^. Upregulation of ERβ increases the sensitivity of non-small cell lung cancer (NSCLC) cells to treatment with doxorubicin and etoposide in p53-defecient NSCLC cells. Mechanistic studies showed that ERβ either enhanced G2–M cell-cycle arrest by activating the checkpoint kinase 1 (Chk1) and altering downstream signaling or induced apoptosis^35^. Our studies also confirmed that ERβ can induce sensitization to chemotherapy in both p53 wild type  and p53 mutant cells and in agreement with the published studies.

Resistance of the chemotherapeutic drug TMZ for GBM treatment is a major clinical issue to patients and the development of an alternative therapy is urgently needed. Recent studies identified that among GBM patients who have received standard of care treatment with surgery, radiation, and TMZ, females exhibited significant survival advantage compared to males^36^. Further, the current standard of care treatment is more effective for female GBM patients than for males and adjuvant TMZ exhibited significant sex differences in therapeutic effects in patients^37^. Our studies indicated that ERβ has the potential to enhance efficacy of TMZ chemotherapy both *in vitro* and *in vivo*. Further, ERβ expression sensitized TMZ resistant GBM cells to TMZ therapy. Our results are supported by earlier published data in other models of cancer. For example, inhibition of ERβ promoted cisplatin resistance by enhancing DNA repair in medulloblastoma cell lines^22^. ERβ expression also has altered the chemo sensitivity of endocrine-resistant cells including their response to tamoxifen therapy^20^. Collectively, these results further support that ERβ mediated tumor suppressor functions also involve sensitization of GBM cells to chemotherapy.

In conclusion, our data has demonstrated that ERβ mediated tumor suppression involve modulation of multiple pathways including DNA damage response pathways. Further, our studies implicate that the upregulation of ERβ1 in conjunction with chemotherapy is a viable and promising therapy for GBM.

## Materials and Methods

### Cell lines and reagents

Human GBM cells U87, U251 and T98G were obtained from American Type Culture Collection (ATCC, Manassas, VA) and were cultured as per ATCC guidelines. Cells were used from early passages (less than 10 passages after thawing). Generation and characterization of ERβ overexpressing and KO cells was described in earlier publication^19^ Generation of primary GBM line GBM-040815 was earlier described^19^ and cultured in neurobasal medium supplemented with B27 serum-free supplement, EGF (20 ng/mL), bFGF (20 ng/mL), LIF (10 ng/mL) and heparin (5 µg/mL). Primary GBM cells were established from discarded patient specimens using UT Health San Antonio Institutional Review Board (IRB) approved protocol. These specimens were de identified and no clinical linkers or codes were accessible to the PI or research personnel. All the methods involving human tissue were conducted in accordance with the declaration of Helsinki and the standards defined by UTHSA Institutional Review Board. Following standard laboratory protocols, all study model cells utilized were determined to be free of mycoplasma contamination and were confirmed by using Mycoplasma PCR Detection Kit acquired from Sigma (St. Louis, MO). Short tandem repeat polymorphism analysis (STR) of the cells was used to confirm the identity using University of Texas Health San Antonio (UTHSA) core facilities. The β-actin (Cat#A-2066) and FLAG (Cat # F3165) antibodies were procured from Sigma. p-ATM (Cat #4526), p-H2AX (Cat # 9718), H2AX (Cat # 7631), cleaved caspase3 (Cat # 9661) antibodies were bought from Cell Signaling Technology (Beverly, MA). ATM antibody (Cat # A300-299A) was purchased from Bethyl laboratories. Validated ERβ monoclonal antibody^38^ (Cat #PPZ0506-00) was purchased from Perseus proteomics (Tokyo, Japan).

### Cell viability and clonogenic assays

Using MTT assay, the effect of ERβ on GBM cell viability was analyzed, as described previously^18^. In 96-well plates, GBM cells (1 × 10^3^ cells/well) were seeded and incubated overnight, after which cells were treated with varying doses of TMZ, lomustine, cisplatin and carboplatin. Then, cell viability was measured after five days. For clonogenic survival assays, GBM cells were seeded in triplicates in 6 well plates (500 cells/well) and after 12 h cells were treated with TMZ for 3 days, and after 2 weeks, colonies that contain ≥50 cells were counted and used in the analysis.

### Cell lysis and Western blotting

Total cell lysates were prepared using modified RIPA buffer (150 mM NaCl, 50 mM Tris-HCl, 50 mM NaF, 5 mM EDTA, 0.5% [wt/vol] sodium deoxycholate and 1% Triton X-100) comprising of phosphatase and protease inhibitors. Lysates were run on SDS-PAGE followed by Western blotting using indicated antibodies and developed using the ECL methodology.

### HR reporter assay

HR repair assays were performed using the U2OS-DR-GFP reporter cell line (obtained from Dr. Maria Jasin, Memorial Sloan-Kettering Cancer Center) as described previously^25^. U2OS-DR-GFP cells were transfected stably with an empty vector or Flag-ERβ vector. pCBA-I-SceI expression plasmid was then introduced into these model cells using electroporation. GFP positive cells were analyzed by FACS 72 h later.

### RNA-sequencing and RT-qPCR

The effect of ERβ on global transcriptome was determined by RNA-sequencing as described previously^18^. Total RNA from U87 cells expressing empty vector or Flag-ERβ vector was prepared using RNeasy mini kit (Qiagen, Valencia, CA). Illumina TruSeq RNA Sample preparation and sequencing was done using UT Health San Antonio sequencing core protocol. DEseq was used to identify differentially expressed genes and significant genes with fold change >1.5 and adjusted p value < 0.05 were used in Ingenuity Pathway Analysis (IPA) for interpreting the biological pathways. RNA-seq data has been deposited in the GEO database under a GEO accession number GSE121332. SuperScript III First-Strand synthesis kit (Invitrogen, Carlsbad, CA) was used for Reverse transcription (RT) reactions. RT-qPCR was performed using SYBR Green (Applied Biosystems) on an Illumina Real-Time PCR system using gene-specific qPCR primer sequences obtained from Harvard Primer Bank (http://pga.mgh.harvard.edu/primerbank/). GAPDH transcript levels were used for normalization and the difference in fold expression was calculated by using delta-delta-CT method.

### ***In vivo*** orthotopic tumor models

After obtaining UT Health San Antonio IACUC approval, all animal experiments were performed in accordance to IACUC standards and approved protocol. All methods were developed and performed in accordance to standard of care practices and guidelines set forth by the IACUC as well as all regulatory agencies. Male athymic nude mice between 8 and 10 weeks of age were obtained from Charles River (Wilmington, MO). After labelling with the GFP-Luciferase reporter, 1 × 10^6^ U87 empty vector, or U87-FlagERβ cells, and 5 × 10^5^ T98G empty vector, or T98G ERβ-GFP cells were injected orthotopically in the right cerebrum using established protocol^18^. U87 tumor bearing mice were treated with either a vehicle (control) or TMZ (at a dose of 10 mg/kg body weight) in 1:1 Ora-Plus and Ora-Sweet mixture on day 17, 19, 21, 23 and 25 after tumor cells implantation (7 mice/treatment group). For T98G tumor bearing mice TMZ was given at a dose of 50 mg/kg body weight on day 17, 19, 21, 23 and 25 after tumor cell implantation (5 mice/treatment group). Investigators were not blinded in the animal studies. Mouse survival was determined using GraphPad Prism 6 software (San Diego, CA) in which Kaplan-Meier survival curves and log-rank test were used.

### Immunohistochemistry (IHC) and confocal microscopy

IHC was performed in accordance to the established protocol as described previously^19^. Briefly, we incubated tumor sections with γH2AX and cleaved caspase3 antibodies for overnight at 4 °C. This was then followed by a secondary antibody incubation at room temperature for 45 minutes. We visualized immunoreactivity using DAB substrate and counterstained with hematoxylin (Vector Lab). For confocal analysis, U251-EV, or U251-ERβ cells were cultured on glass coverslips and treated with vehicle or TMZ for 48 h and 72 h. After, cells were fixed in 3.7% paraformaldehyde followed by, permeabilization with 0.1% TritonX-100 for 10 min. Cells were then stained with γH2AX antibody and the fluorescence was analyzed by confocal microscopy.

### Statistical analyses

Using GraphPad Prism 6 software, we analyzed statistical differences by unpaired Student’s t-test and one-way ANOVA. Log-rank (Kaplan–Meier) test was analyzed using GraphPad Prism. All the data are characterized in plots are shown as means ± SE. Statistically significant data consisted in a value of p < 0.05. All *in vitro* assays were performed in triplicate and repeated at least three times.

## Data Availability

All the data generated and/or analyzed during the current study are included in this article and are available from the corresponding author on reasonable request
